# Downregulation of ETS Rescues Diabetes-Induced Reduction of Endothelial Progenitor Cells

**DOI:** 10.1371/journal.pone.0004529

**Published:** 2009-02-19

**Authors:** Florian Hartmut Seeger, Linping Chen, Ioakim Spyridopoulos, Joachim Altschmied, Alexandra Aicher, Judith Haendeler

**Affiliations:** 1 Molecular Cardiology, Department of Internal Medicine III, University of Frankfurt, Frankfurt, Germany; 2 Cell Biology & Molecular Aging Research, IUF (Institut für Umweltmedizinische Forschung) at the University of Duesseldorf gGmbH, Duesseldorf, Germany; Istituto Dermopatico dell'Immacolata, Italy

## Abstract

**Background:**

Transplantation of vasculogenic progenitor cells (VPC) improves neovascularization after ischemia. However, patients with type 2 diabetes mellitus show a reduced VPC number and impaired functional activity. Previously, we demonstrated that p38 kinase inhibition prevents the negative effects of glucose on VPC number by increasing proliferation and differentiation towards the endothelial lineage in vitro. Moreover, the functional capacity of progenitor cells is reduced in a mouse model of metabolic syndrome including type 2 diabetes (Lepr^db^) in vivo.

**Findings:**

The aim of this study was to elucidate the underlying signalling mechanisms in vitro and in vivo. Therefore, we performed DNA-protein binding arrays in the bone marrow of mice with metabolic syndrome, in blood-derived progenitor cells of diabetic patients as well as in VPC ex vivo treated with high levels of glucose. The transcriptional activation of ETS transcription factors was increased in all samples analyzed. Downregulation of ETS1 expression by siRNA abrogated the reduction of VPC number induced by high-glucose treatment. In addition, we observed a concomitant suppression of the non-endothelial ETS-target genes matrix metalloproteinase 9 (MMP9) and CD115 upon short term lentiviral delivery of ETS-specific shRNAs. Long term inhibition of ETS expression by lentiviral infection increased the number of cells with the endothelial markers CD144 and CD105.

**Conclusion:**

These data demonstrate that diabetes leads to dysregulated activation of ETS, which blocks the functional activity of progenitor cells and their commitment towards the endothelial cell lineage.

## Introduction

Postnatal vessel formation, known as angiogenesis, was attributed to the migration and proliferation of preexisting mature endothelial cells [1], [2]. Recent studies demonstrated that circulating bone marrow-derived endothelial progenitor cells contribute to adult blood vessel formation, a process characterized as vasculogenesis [3]. Endothelial progenitor cells play a major role in repair processes after myocardial infarction (reviewed in [4]) and wound healing [5]. However, in vivo and in vitro studies revealed that endothelial progenitor cells from patients with type 1 and type 2 diabetes were impaired in number and function [5]–[8].

The molecular mechanisms underlying the reduced endothelial progenitor cell number and function by high glucose are not yet clearly defined. Recently, Marchetti et al. demonstrated that high glucose levels (33 mM) increased apoptosis of endothelial progenitor cells in vitro and impaired the PI3-kinase/Akt pathway [9]. In addition, activation of Akt signalling stimulates the proliferation and inhibits apoptosis of endothelial progenitor cells cultured under high-glucose conditions and importantly increases ischemia-induced mobilization of endothelial progenitor cells [10]. Our previous study showed that high glucose levels (15 mM) did not alter apoptosis in endothelial progenitor cells in vitro, but activated p38 MAP kinase and several downstream targets such as the transcription factor CREB. Moreover, we found that high glucose levels decreased endothelial progenitor cell proliferation and differentiation towards the endothelial cell lineage in vitro [11]. Unfortunately, no animal studies in models for type 2 diabetes or metabolic syndrome were conducted to identify signalling mechanisms in vivo which contribute to high glucose-induced impairment of progenitor cells. One critical issue is to choose an appropriate mouse model that closely resembles type 2 diabetes in humans, which is the major form of diabetes and accounts for 95% of diabetes cases [12]. There are very few animal studies to identify signalling mechanisms of endothelial progenitor cells in diabetes. It was shown that NO-mediated impaired mobilization may be responsible for the progenitor cell reduction in diabetic mice [13]. However, Gallagher et al. used streptozocin-treated mice, a model not comparable with type 2 diabetes in humans. To study type 2 diabetes in a mouse model we chose the leptin receptor knock out mouse model (Lepr^db^). Lepr^db^ mice lack functional leptin receptors, become obese shortly after birth and are insulin resistant and hyperglycemic as adults [14].

The aim of this study was to investigate potential signalling mechanisms in vasculogenic progenitor cells (VPC), which regulate the differentiation processes in vitro and in vivo.

ETS (E26 transformation-specific DNA binding domain) is one of the largest families of transcriptional regulators that share a highly conserved DNA-binding domain (ETS domain). ETS transcription factors are downstream of the p38 MAP kinase, which has already been shown to be important for endothelial progenitor cell differentiation and proliferation [11]. As ETS transcription factors are important for proliferation, survival and differentiation, we hypothesized that this could also be an important mechanism in VPC (for review see [15]).

We found that ETS DNA-binding activity is much higher in diabetic cells. Inhibition of the ETS activity leads to increase in impaired VPC number. Therefore this could be a potential mechanism to improve the impaired VPC number in patients with type 2 diabetes.

## Methods

### Study population and patient characteristics

Mononuclear cells (MNC) were isolated from fresh peripheral blood of healthy human volunteers or patients with type 2 diabetes.

Written informed consent was obtained from all patients. The ethics review board of the Hospital of the Johann Wolfgang Goethe University of Frankfurt, Germany, approved the protocol, and the study was conducted in accordance with the Declaration of Helsinki.

### Mice

Leptin-receptor-deficient mice (Lepr^db^ mice) were obtained from Charles River Laboratories and used for the experiments at an average age of 12 weeks.

The animal studies were approved by the “Regierungspräsidium” Darmstadt, Germany. All experiments were carried out in accordance with the “Position of the American Heart Association on Research Animal Use”.

### Isolation and Cultivation of VPC

MNC were isolated by density gradient centrifugation with Biocoll separating solution (Cambrex; 800×g, 20 min, without brake) from fresh peripheral blood of healthy human volunteers or patients with type 2 diabetes as previously described [11]. MNCs (8×10^6^ cells/ml medium) were plated on culture dishes coated with human fibronectin (Sigma) and maintained in endothelial basal medium (EBM; CellSystems) supplemented with 1 µg/ml hydrocortisone, 12 µg/ml bovine brain extract, 50 µg/ml gentamycin, 50 ng/ml amphotericin B, 10 ng/ml epidermal growth factor and 20% FCS. After 3 days of culture, more than 90% of the VPC express endothelial marker proteins KDR, VE-Cadherin and von Willebrand factor [16]–[18]. Dual-staining cells positive for both lectin and 1,1′–dioctadecyl–3,3,3′,3′–tetramethylindocarbocyanine–labelled acetylated low–density lipoprotein (Dil-Ac-LDL) (Harbor Bio-Products) were judged as VPC [16]. VPC have the capacity of tube formation in vitro and improve blood flow in murine models of hindlimb ischemia [19]. Dendritic cell markers such as CD1a or CD83 were not expressed, however, being early endothelial progenitors, these cells still bear myeloid and hematopoietic features, which are lost during further culture into late outgrowth colonies. Since there is an ongoing debate how to define endothelial progenitors [20], in particular whether the expression of hematopoietic lineage-specific markers such as CD45 precludes endothelial progenitor progeny [21], we decided to use the term ‘vasculogenic’ (VPC) instead of ‘endothelial’ progenitor cells (EPC) to more emphasize the functional capacity of these cells than their origin. In some experiments, MNC were incubated with glucose (Braun Melsungen), mannitol served as osmotic control. After 3 days in culture, adherent cells were incubated with Dil-Ac-LDL for 1 hour and 4–5 random power-fields were counted by independent investigators and with a computer-based program.

### Isolation of Bone Marrow Mononuclear Cells

Bone marrow of animals was harvested by flushing femurs and tibias with PBS. Mononuclear cells (BMC) were isolated by density gradient centrifugation using Ficoll (Cambrex; 800×g, 20 min, without brake). BMC were washed three times with PBS (800×g), counted and used for the experiments.

### Production of lentiviral particles

In order to achieve long term gene silencing, DNA-oligonucleotides encoding shRNAs directed against ets1 or ets1 and ets2 were subcloned into pLKO.1-puro (Sigma). Lentiviral particles were produced in 293T packaging cells using a calcium phosphate co-precipitation method.

### Infection of human MNC

The lentiviral particles were added (1∶5) to MNC immediately after isolation. A second round of infection was performed the next day and RNA was isolated on day 4. For long term experiments the number of adherent VPC was determined after staining with PE-conjugated antibodies against CD105 (BD Biosciences) and CD144 (BD Biosciences) at day 14 after infection. The biosafety level 2 experiments were approved by the “Bezirksregierung” Düsseldorf.

### Protein-DNA binding array

After isolation of cells, nuclear and cytosolic fractions were separated using a commercially available kit according to the manufacturer's protocol (Pierce). Nuclear and cytosolic fractions were controlled for purity in Western blots with antibodies directed against the exclusively nuclear or cytosolic proteins Topoisomerase I (nuclear) and Hsp70 (cytosolic) as described previously [22]. Protein/DNA arrays were performed according to the manufacturer's protocol (Panomics, USA). Briefly, 25 µg of nuclear extract was incubated with the biotinylated TranSignal® Probe Mix. DNA/protein complexes were washed. Then, DNA was separated from protein and hybridized on the membranes at 42°C. The bound biotinylated DNA probes were detected using streptavidin coupled horseradish peroxidase and a luminogenic peroxidase substrate (ECL). Signals were detected using ECL-Hyperfilm (Amersham, Germany) and semiquantitative analysis was performed using the Tina-software 2.09 (Raytec). As suggested by the manufacturer the local area surrounding the individual spots was used for background subtraction. The membranes contain prespotted binding sites for various transcription factors in duplicate and two different dilutions annotated by the manufacturer, three of which belong to the ETS family (ETS, ETS/PEA3, EGR). According to genecards (www.genecards.org) these proteins are defined as follows: ETS: v-ets erythroblastosis virus E26 oncogene homolog (avian), ETS/PEA3: ets variant gene 4 (ETV4), EGR: early growth response. “ECL spots” are positive controls on the membrane and, according to the manufacturers specifications, can be used to normalize signal intensities. Therefore, after calculating the density of the spots using Tina software, the membranes were normalized to the average of their “ECL spot density” (average of all ECL spots). Only binding sites were the densitometric analysis of duplicate spots and the corresponding dilutions yielded the same results were analyzed in more detail.

### Real-time RT(reverse transcriptase)-PCR

Gene-specific mRNA expression was determined by real-time RT-PCR. RNA concentration was determined photometrically and aliquots of total RNA (2 µg) were used for cDNA-synthesis with the SuperscriptTM III First-Strand synthesis system for RT-PCR (Invitrogen, Karlsruhe, Germany). PCR reactions were carried out on an Abi Prism 7000 (Applied Biosystems, Foster City, CA, USA) using SYBRGreen PCR Master Mix (Applied Biosystems, Darmstadt, Germany). For relative expression comparison between different cells the ΔΔCT method was used, utilizing the 18S RNA as housekeeping gene. Primers were designed using the online-software Primer3. The following primer pairs were used:

18S RNA: GCCGCTAGAGGTGAAATTCTTG, CATTCTTGGCAAATGCTTTCG
CD105: CAACTGTGTGAGCCTGCTGT, GACAGGTCAGGGCTGATGAT
CD115: ATGGTCATGTGGCCAAGATT, GAGGATGCCATAGGACCAGA
ETS1: GAGTCAACCCAGCCTATCCA, CCGAGGGGTAGTCATTCTCA
ETS2: TCTGGTGAACGTGAATCTGC, CGGAGGTGAGGTGTGAATTT
MMP9: TTGACAGCGACAAGAAGTGG, GCCATTCACGTCGTCCTTAT

### Statistical analysis

Data are expressed as mean±SEM from at least 3 independent experiments unless stated otherwise. For in vitro data, statistical analysis was performed by a two-sided student *t* test. Statistical significance was assumed at a value of p<0.05.

## Results

### Induced activation of ETS transcription factors in Lepr^db^ mice

**Figure 1:**
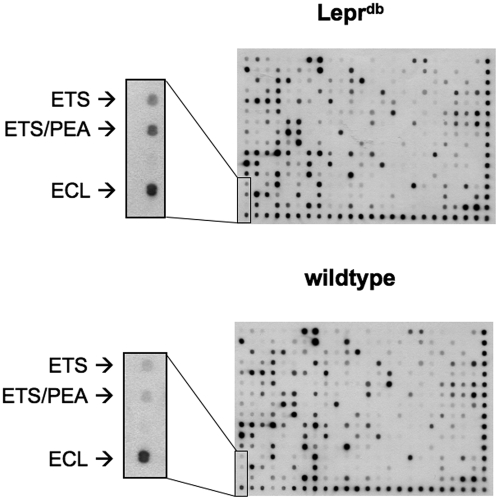
ETS-DNA-protein binding activity in BMC of Lepr^db^ mice. DNA-binding activity of ETS family proteins in BMC of Lepr^db^ mice and their age-matched littermates was analyzed by the use of precoated membranes (Panomics). In all cases 25 µg of nuclear extracts were used. Representative membranes of n = 3 experiments are shown.

Because diabetes leads to impairment in number and functional activity of progenitor cells in patients [7], [8], [16], we wanted to elucidate the potential underlying signalling mechanisms. Therefore, we performed DNA-protein binding arrays with bone marrow-derived mononuclear cells from Lepr^db^ mice and compared them to their wild type littermates. Out of 450 transcription factors, the activity of ETS transcription factors was significantly increased in BMC of Lepr^db^ mice compared to the wild type littermates (Figure 1). Similarly, we found an increase in ETS1 protein expression in BMC of Lepr^db^ mice (data not shown).

### Activation of ETS transcription factors under hyperglycemic conditions in vitro as well as in patients with type 2 diabetes

**Figure 2:**
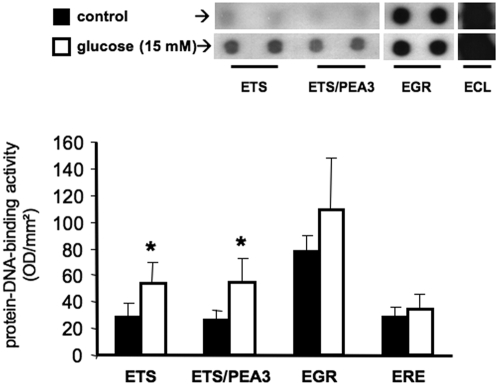
ETS-family DNA-binding activity in VPC after ex vivo glucose stimulation. Mononuclear cells were cultivated in EBM as described in the presence or absence of 15 mM glucose. DNA binding of different transcription factors was analyzed by the use of precoated membranes (Panomics). In all cases 25 µg of nuclear extracts were used. Representative membranes of n = 3 experiments are shown, *p<0.05 vs. control.

**Figure 3:**
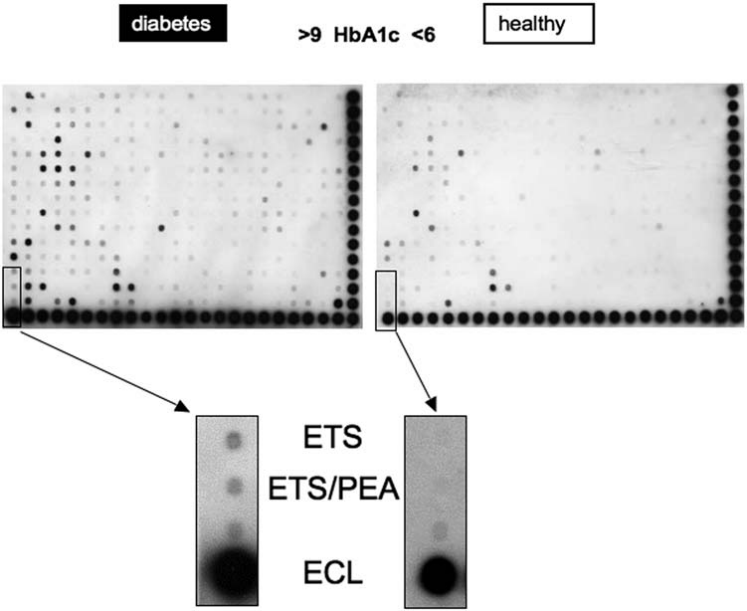
ETS in VPC of subjects with type 2 diabetes. DNA-binding activity of different transcription factors in ex vivo cultivated VPC of healthy controls and patients with type 2 diabetes was analyzed by the use of precoated membranes (Panomics). In all cases 25 µg of nuclear extracts were used. Representative membranes of n = 3 experiments are shown.

To further investigate that protein-DNA-binding activity of ETS transcription factors occurs also in humans, we first isolated human peripheral blood mononuclear cells and cultivated the cells for 4 days in the presence or absence of high glucose (15 mM). DNA-protein binding arrays were performed and, similar as in Lepr^db^ mice, ETS transcription factors were activated under high glucose culturing conditions (Figure 2). Having demonstrated that high glucose enhanced DNA-binding activity of ETS transcription factors, we next examined ETS DNA-binding activity in peripheral blood-derived VPC from healthy volunteers as well as patients with type 2 diabetes (for characteristics see table 1). Indeed, ETS-DNA binding activity is increased in patients with type 2 diabetes compared to healthy volunteers (Figure 3). Of note, this difference was obtained after 3 days of culture under similar conditions.

**Table 1:** 

	Healthy	Diabetes	p-value
	(n = 4)	(n = 4)	
**age (years)**	56±5	62±16	n.s.
**gender (% male)**	50	50	n.s.
**hypertension (%)**	0	75	p<0.05
**smoker (%)**	25	25	n.s.
**diabetes (%)**	0	100	p<0.05
**insulin (%)**		50	
**oral therapy (%)**		50	
**HbA1c (%)**	5.8±0.1	7.5±1.0	p<0.05

### Ablation of ETS1 expression by siRNA rescued high glucose-induced reduction of progenitor cell number

**Figure 4:**
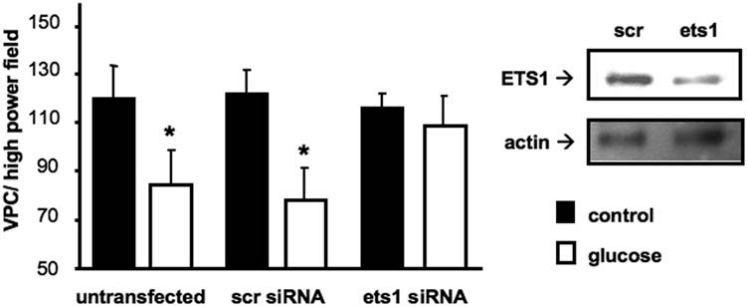
ETS1 knockdown counteracts high-glucose induced reduction of VPC numbers. Mononuclear cells were transfected with ETS1-specific siRNA (ets1), a scrambled control (scr) at day 0 or left untransfected and stimulated with glucose. After 3 days of culture, VPC were stained and counted by independent investigators (left side). Data are mean±SEM of n≥6; *p<0.05 vs. control. ETS1 protein expression was examined by immunoblot analysis (right side). Actin was used as loading control.

To demonstrate a causal link between ETS transcription factors and the reduced number of progenitor cells in type 2 diabetes, we transfected peripheral blood-derived mononuclear cells with control siRNA or siRNA against ETS1 and incubated them with high glucose for 3 days. High glucose levels induced a significant reduction of adherent VPC when transfected with control siRNA (Figure 4). In contrast, in cells transfected with ets1 siRNA the negative effect of high glucose levels on VPC number was abrogated (Figure 4).

### Downregulation of ETS transcription factors suppressed non-endothelial ETS-target genes MMP9 and CD115 and increased long term endothelial lineage commitment of VPC

**Figure 5:**
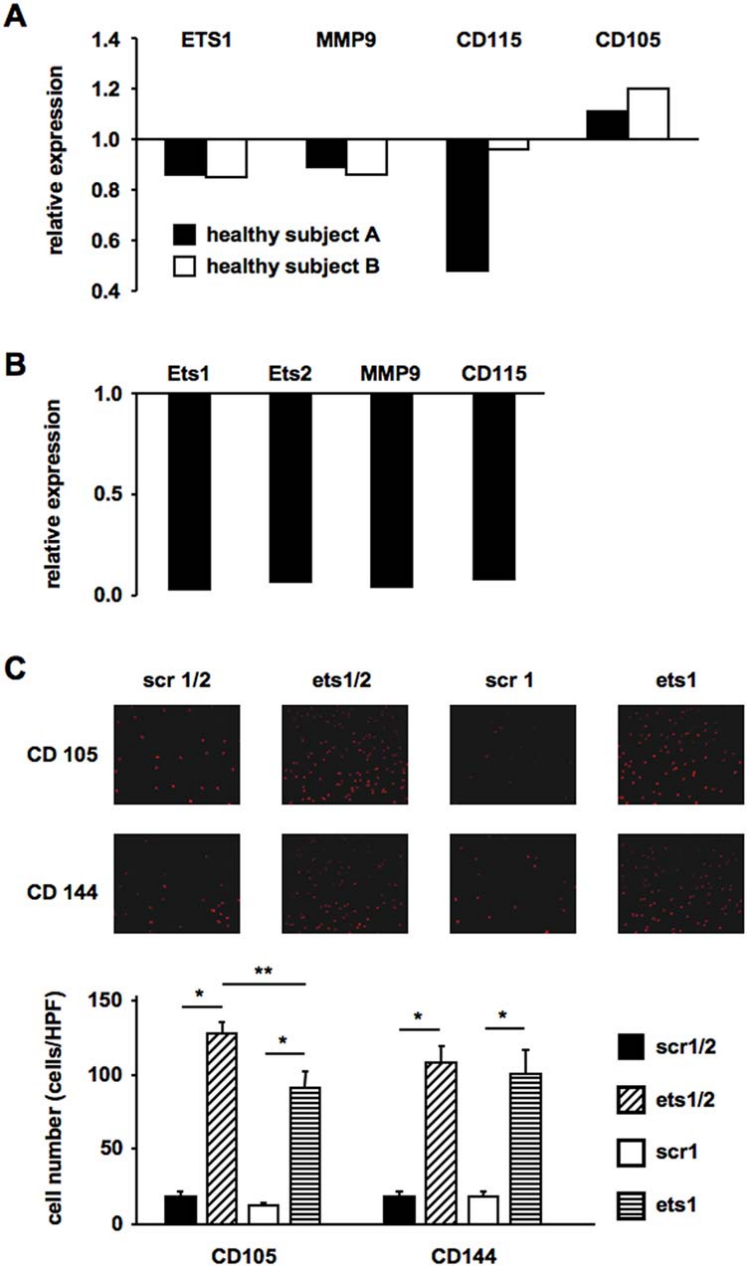
ETS knockdown suppresses expression of non-endothelial ETS target genes and induces CD105 and CD144. Mononuclear cells were infected with lentiviruses expressing shRNAs targeting ETS1 (ets1) or ETS1 and ETS2 (ets1/2) or the corresponding scrambled shRNA controls (scr1, scr1/2). (A+B) After 4 days of culture RNA was prepared, and real-time RT-PCR was performed. Expression was normalized to 18S RNA and is shown relative to RNA isolated from cells transduced with a virus expressing the corresponding scrambled shRNA (scr). (A) ets1 knockdown, (B) ets1/2 knockdown. (C) After 12 days of culture, adherent VPC were stained with CD105 and CD144 antibodies and positive cells were counted. The upper part shows representative images, the lower part shows the quantification of the data of n = 4 experiments. Data are mean±SEM. *p<0.05 vs. scr, **p<0.05 vs. ets1.

In the next set of experiments, we wanted to elucidate whether knock down of ETS transcription factors has an influence on the differentiation capacity of MNC. Therefore, we designed lentiviral shRNA vectors to permanently knock down ETS1 or ETS1 and ETS2 expression. Indeed, short term lentiviral knockdown of ETS1 expression resulted in a concomitant suppression of the known non-endothelial ETS-target genes MMP9 and CD115 [23] and an increase in the endothelial marker CD105 (Figure 5A). Knockdown with a more potent shRNA virus targeting ETS1 and ETS2 completely abolished expression of MMP9 and CD115 (Figure 5B). Strikingly, knockdown of ETS transcription factors over two weeks led to a stong increase in CD105- and CD144-positive adherent cells (Figure 5C). The effect on CD105 positive cells was even more pronounced, when we knocked down both ETS1 and ETS2, clearly demonstrating a shift towards endothelial lineage commitment of peripheral blood derived mononuclear cells.

## Discussion

The data of the present study demonstrate that increased nuclear DNA-binding activities of ETS1 and ETS2 transcription factors are crucial determinants of diabetes- and high glucose-induced reduced number of VPC. Moreover, increased DNA-binding activity of ETS-transcription factors inhibits the commitment of progenitor cells towards the endothelial cell lineage.

Emerging evidence indicates that endothelial progenitor cells are involved in the maintenance of vascular homeostasis and their impairment may be conducive to vascular disease. Endothelial progenitor cell function is impaired in patients with coronary artery disease and diabetes [7], [16]. Recently, Fadini et al. suggested that diabetes is the most relevant risk factor associated with a reduction of endothelial progenitor cells [5], [6]. However, the underlying mechanisms are still not understood. Marchetti et al investigated the effects of high glucose toxicity (33 mM) on endothelial progenitor cells and determined that high glucose reduced phosphorylation of Akt and thereby increased activation and nuclear localization of the forkhead transcription factor-1 previously shown to have negative impact on endothelial function [24]. Our study now provides evidence that in vivo and in vitro the number of VPC is reduced in conditions of type 2 diabetes and that one major signalling pathway involved is the activation of the transcription factors ETS1 and ETS2.

The ETS protein family is a group of transcription factors that share a DNA-binding ETS domain and regulate the expression of a variety of genes including key ones involved in regulation of cell proliferation, differentiation and survival. Because of their critical role in basic cellular processes, dysregulation of ETS transcription factors can be found in many human diseases with the need for neovascularisation, such as cancer. ETS transcription factors have been implicated in the regulation of genes involved in homeostasis, vascular development and angiogenesis [15], even though no endothelial specific ETS transcription factor has been identified by now.

We could clearly show that ETS dysregulation by high levels of glucose leads to impairment of progenitor cell number as well as functional capacity. The importance of circulating endothelial progenitor cell number, measured as CD34^+^KDR^+^ cells in the peripheral blood, has recently been shown as these cells predict the outcome in CAD-patients [25], [26].

Although the transcription factor ETS reduces VPC numbers, ETS activity is clearly important in adult angiogenesis [27], [28]. In the resting endothelium, ETS1 is expressed at a very low level. During angiogenesis, re-endothelialisation after balloon denudation in a rat model as well as in scratch wound migration assays, ETS1 is transiently expressed at high levels in endothelial cells [29], [30], suggesting that during the process of vessel formation or repair upregulation of ETS1 transcription factor expression is required in mature endothelial cells. However, the process of preexisting mature endothelial cells to perform angiogenesis differs completely from VPC-induced vasculogenesis or vascular repair of the damaged endothelial layer.

Even though our findings demonstrate the importance of ETS1 in angiogenesis, ETS1 deficient mice develop normally except for an increased perinatal mortality [31], [32]. Especially no vascular phenotype can be detected. Therefore, we hypothesized that most likely other ETS transcription factors can compensate for its loss. Alternatively, these mice might have increased numbers and improved functionality of their endothelial progenitor cells for compensation.

Members of the ETS gene family are known to be expressed in the hematopoietic tissue and some of them play a pivotal role in hematopoietic cell development. The special importance of ETS1 regulation in differentiation of hematopoietic stem cells has previously been shown. During erythroid differentiation, ETS1 is downregulated and exported out of the nucleus. In contrast, during megakaryopoiesis, ETS1 increases and remains in the nucleus [33]–[36]. Similarly, it seems that ETS1 activation is required for angiogenesis by sprouting of mature endothelial cells and down-regulation of ETS DNA binding activity is required for increased VPC differentiation from their progenitors as shown in this study.

We clearly showed that glucose upregulates ETS DNA binding activity and thereby reduces VPC numbers. In addition, it has been shown that other pro-inflammatory stimuli/cardiovascular risk factors such as TNFα up-regulate ETS transcription factors [37] and reduce the number of endothelial progenitor cells [11]. Therefore, it is tempting to speculate that not only high glucose but also TNFα prevents endothelial cell lineage commitment by ETS transcriptions factor activation.

Taken together, the present study demonstrates a critical role for ETS transcription factors on VPC number and function. In vitro and in vivo high glucose levels increased ETS DNA-binding and thus most likely transcriptional activity. Inhibition of ETS1 and ETS2 expression counteracts the reduction of VPC number by enhancing endothelial lineage commitment. Given the fact that ETS transcription factors regulate a plethora of genes [23], a systematic analysis of other downstream targets besides CD115, MMP9, CD144 and CD105 investigated here is required to fully understand the role of the ETS family in the cardiovascular system.
